# Giant mediastinal mass

**DOI:** 10.11604/pamj.2020.37.162.26304

**Published:** 2020-10-15

**Authors:** Danilo Coco, Silvana Leanza

**Affiliations:** 1 Department of General Surgery, Ospedali Riuniti Marche Nord, Pesaro, Italy,; 2 Department of General Surgery, Carlo Urbani Hospital, Jesi, Ancona, Italy

**Keywords:** Giant mediastinal mass, mediastinal lymphoma B-cells, computed tomography

## Image in medicine

A 43-year-old Caucasian female presented to the emergency department (ED) with significant dyspnea, thoracic pain and fever. She presented a negative medical history and no therapy. During the physical examination, the patient was uncomfortable. Her vital signs were: blood pressure, 100/90 mmHg; respiratory rate, 50 breaths/minute; heart rate, 130 beats/minute; and temperature superior to 38°C. Oxygen saturation was 80% on room air and 90% with the aid of oxygen. The abdominal examination was unremarkable. Laboratory evaluation revealed high leukocytosis with a white blood cell (WBC) count of 15 per mm^3^. Arterial Blood Gases (ABG) demonstrated respiratory acidosis: PO_2_80, PCO_2_60, HCO_3_30 mEq. Thoracic X-ray revealed a massive pleural effusion. Computed tomography demonstrated a giant mediastinal mass surrounding pulmonary artery, aorta and pericardia pleura associated with massive pleural effusion. The patients immediately started intravenous (IV) fluids of 2l in 6 hours, Foley and jugular catheter vein cannulation to support main arterial pressure and urine output. The patient was transferred to surgical services where a 28 Fr thoracic drainage was inserted. Post-drainage thoracic scan (CT) demonstrated only the giant mediastinal mass. Fine-needle aspiration (FNA) CT scan guided was performed. Histopathological findings were mediastinal lymphoma B-cells. The patient was discharged three days after.

**Figure 1:**
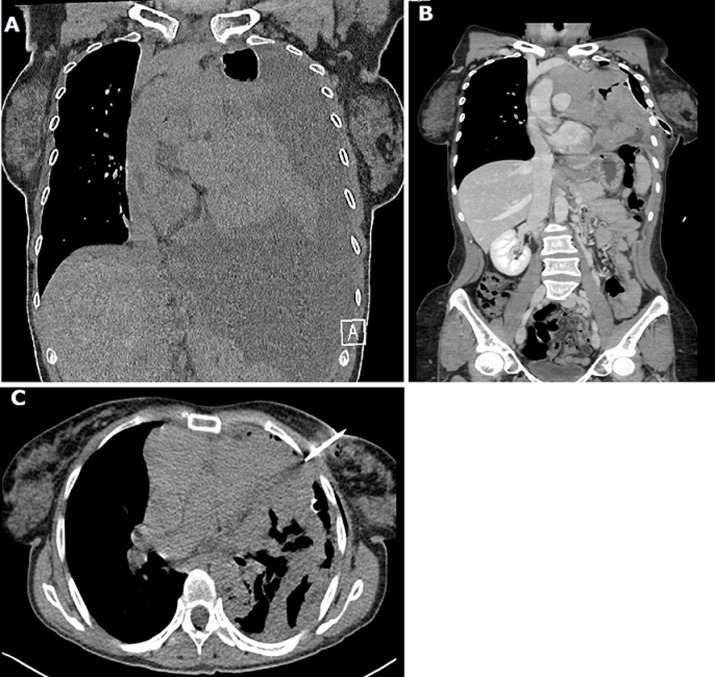
A) thoracic computed tomography (CT) scan demonstrating a giant mediastinal mass associated with massive left pleural effusion; B) post-28 Fr thoracic drainage demonstrating the extension of giant mediastinal mass; C) FNA thoracic CT scan biopsy

